# Supplementary material for: Chimeric Antigen Receptor-Engineered Human Gamma Delta T Cells: Enhanced Cytotoxicity with Retention of Cross Presentation
Source: Mol Ther. 2017 Dec 8;26(2):354–65. doi: 10.1016/j.ymthe.2017.12.001 (PMC5835118; doi:10.1016/j.ymthe.2017.12.001)
Supplement: Document S1. Figures S1–S3 [file mmc1.pdf]

YMTHE, Volume *26*

Supplemental Information

Chimeric Antigen Receptor-Engineered Human Gamma Delta T Cells: Enhanced Cytotoxicity with Retention of Cross Presentation

Anna Capsomidis, Gabriel Benthall, Heleen H. Van Acker, Jonathan Fisher, Anne M. Kramer, Zarah Abeln, Yvonne Majani, Talia Gileadi, Rebecca Wallace, Kenth Gustafsson, Barry Flutter, and John Anderson

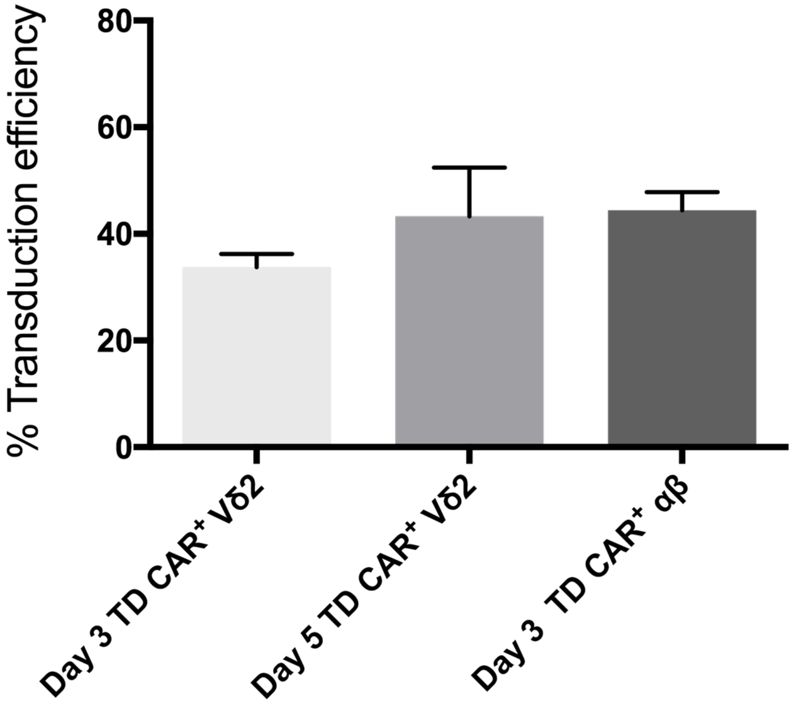

Supplementary Figure 1.
Transduction efficiency of Vδ2⁺ T cells as measured by % QBend10⁺ comparing timing of viral transduction (day 3 or day 5). As a positive control PBMC stimulated by CD3 and CD28 antibodies were transduced on day 3 following stimulation.

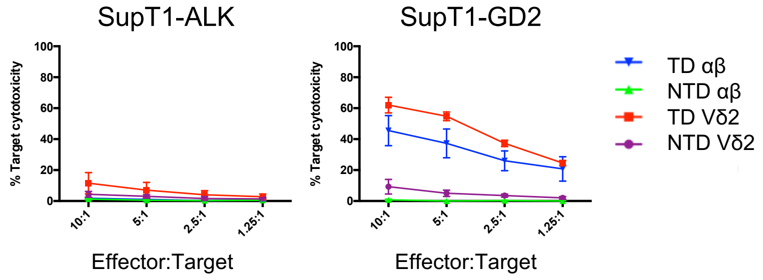

Supplementary Figure 2. αβ and Vδ2⁺ GD2-CAR T cells specifically lyse SupT1 cells genetically modified to express tumor antigen GD2, but not SupT1 cells genetically modified to express anaplastic lymphoma kinase (ALK) antigen in 4-hour ^{51}Cr release assay. GD2-CAR transduced (TD) αβ and Vδ2⁺ T cells were sorted to >95% purity by fluorescence activated cell sorting (FACS). Non-transduced (NTD) cells were sorted by magnetic-activated cell sorting (MACS). Mean of 4 donors in triplicate. Error bars represent SEM.

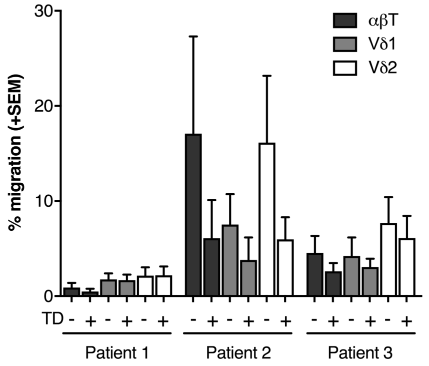

Supplementary Figure 3. Percentage T cell migration towards supernatant taken from three neuroblastoma neurosphere lines derived from primary patient tissue. Migration calculated as ((number of migrated cells in the specific condition - number of migrated cells in the negative control for that condition) / number of migrated cells in the positive control) x 100.
