# Supplementary material for: Chimeric Antigen Receptor-Engineered Human Gamma Delta T Cells: Enhanced Cytotoxicity with Retention of Cross Presentation
Source: Mol Ther. 2017 Dec 8;26(2):354–65. doi: 10.1016/j.ymthe.2017.12.001 (PMC5835118; doi:10.1016/j.ymthe.2017.12.001)
Supplement: Document S2. Article plus Supplemental Information [file mmc2.pdf]

Molecular Therapy
Original Article

Chimeric Antigen Receptor-Engineered Human Gamma Delta T Cells: Enhanced Cytotoxicity with Retention of Cross Presentation

Anna Capsomidis,[1] Gabriel Benthall,[1] Heleen H. Van Acker,[1] Jonathan Fisher,[1] Anne M. Kramer,[2] Zarah Abeln,[2] Yvonne Majani,[1] Talia Gileadi,[1] Rebecca Wallace,[1] Kenth Gustafsson,[2] Barry Flutter,[1] and John Anderson[1]

[1]Cancer Section, University College London Institute of Child Health, London, UK; [2]Molecular and Cellular Immunology Section, University College London Institute of Child Health, London, UK

Gamma delta T (γδT) lymphocytes are primed for rapid function, including cytotoxicity toward cancer cells, and are a component of the immediate stress response. Following activation, they can function as professional antigen-presenting cells. Chimeric antigen receptors (CARs) work by focusing T cell function on defined cell surface tumor antigens and provide essential costimulation for robust activation. Given the natural tropism of γδT cells for the tumor microenvironment, we hypothesized that their transduction with CARs might enhance cytotoxicity while retaining their ability to migrate to tumor and act as antigen-presenting cells to prolong the intratumoral immune response. Using a GD2-targeting CAR as a model system, we showed that γδT cells of both Vδ1 and Vδ2 subsets could be expanded and transduced to sufficient numbers for clinical studies. The CAR added to the cells' innate cytotoxicity by enhancing GD2-specific killing of GD2-expressing cancer cell lines. Migration toward tumor cells *in vitro* was not impaired by the presence of the CAR. Expanded CAR-transduced Vδ2 cells retained the ability to take up tumor antigens and cross presented the processed peptide to responder alpha beta T (αβT) lymphocytes. γδ CAR-T cell products show promise for evaluation in clinical studies of solid tumors.

INTRODUCTION

Adoptive cellular therapy with T cells engineered to express chimeric antigen receptors (CARs) combines the antigen specificity of a monoclonal antibody with potent T cell activation, proliferative potential, and cytotoxic function. CARs comprise an extracellular antigen-binding domain, most commonly a single-chain variable fragment (ScFv), combined with a transmembrane domain and one or more intracellular signaling domains. First-generation CARs typically had a single CD3-ζ intracellular signaling domain; they were shown to have poor persistence *in vivo*.[1] Second-generation and third-generation CARs were created by the addition of one or two costimulatory endodomains to the CD3-ζ motif to provide tailored signals with the aim of enhancing activation and survival. Current costimulatory endodomain candidates include CD27, CD28, 41BB, ICOS, and OX40. Though CD19-specific CAR-T cells have shown impressive responses in patients with B cell hematological malignancies, in some cases leading to complete remission of chemotherapy refractory disease,[2] evaluation of CAR-T cells in solid tumors has so far been less successful.[3] For example, a phase 1 trial using first-generation GD2-CAR-T cells in patients with neuroblastoma demonstrated some objective clinical responses.[4,5] However, a follow-up study with third-generation CAR has shown no evidence of enhanced clinical efficacy despite markedly enhanced cytokine secretion and proliferation *in vitro*.[6] We speculate that the poor responses against solid tumors might be due to the immunosuppressive tumor microenvironment impairing T cell homing, cytotoxicity, and survival. Thus, alternative approaches, including the use of unconventional lymphocytes that might be naturally tumoricidal and capable of enhanced tumor trafficking, should be evaluated.[7] Gamma delta T (γδT) cells are particularly intriguing given their capacity to differentiate following activation into cells with professional antigen presentation function.

γδT cells comprise 1%–5% of circulating T cells but are the predominant lymphocyte at epithelial surfaces.[8] A meta-analysis of gene expression data from more than 18,000 cancers identified infiltration by γδT cells to be the most significant factor associated with favorable prognosis.[9] γδT cells are a heterogeneous population characterized by expression of Vγ(2–5, 8, and 9) and Vδ(1–8) chains to form a heterodimeric γδT cell receptor (TCR). γδT cells of the Vγ9Vδ2 subtype are predominant in circulating peripheral blood and can be selectively expanded *in vitro* and *in vivo* to a clinically significant number with zoledronate (ZOL), an aminobisphosphonate drug used in clinical practice to treat osteoporosis and bony metastatic disease.[10] ZOL inhibits farnesyl pyrophosphate synthase, an enzyme in the mevalonate pathway of cholesterol biosynthesis. This leads to an accumulation of upstream metabolites

Received 22 August 2017; accepted 2 December 2017;
https://doi.org/10.1016/j.ymthe.2017.12.001.

Correspondence: Barry Flutter, Cancer Section, University College London Institute of Child Health, 30 Guilford Street, London, WC1N 1EH, UK.
E-mail: b.flutter@ucl.ac.uk
Correspondence: John Anderson, Cancer Section, University College London Institute of Child Health, 30 Guilford Street, London, WC1N 1EH, UK.
E-mail: j.anderson@ucl.ac.uk

This is an open access article under the CC BY license (http://creativecommons.org/licenses/by/4.0/).

including isopentenyl pyrophosphate, resulting in activation and proliferation.[11] Vγ9Vδ2 cells have endogenous cytotoxicity against various tumors;[12] following activation, they can acquire phenotypic characteristics of professional antigen-presenting cells (γδ-APCs), including capacity for cross presentation of tumor-associated antigens.[13–16] γδT cells of the Vδ1 subtype are also of potential clinical interest due to their naturally more naive memory (T_{naive}) phenotype,[17] a reduced susceptibility to activation-induced cell death,[18] and their natural residency in tissues. We and others have shown that this subclass can be expanded from peripheral blood to clinically significant numbers using artificial APCs,[19,20] T cell mitogens such concanavalin A (ConA),[21] or anti-CD3 antibody.[22]

Previous studies have described the feasibility of viral transduction[23] or electroporation[20] of γδT cells with CARs. However, the relative functionality of engineered CAR^+ γδT cells compared with conventional adoptive CAR^+ T cell approaches has yet to be fully characterized, and large-scale manufacturing protocols for adoptive T cell transfer of CAR^+ γδT cells have yet to be developed. Here we describe, using a GD2 antigen model against a range of GD2-expressing cells, an approach for the transduction and expansion of CAR^+ γδT cells from peripheral blood to sufficient numbers for adoptive T cell transfer. We also demonstrate the acquisition of both CAR-dependent antigen-specific killing and antigen cross-presentation function.

RESULTS

ZOL and ConA Activation Result in Preferential Expansion of γδT Cells from Peripheral Blood

To evaluate a potential role of human peripheral blood γδT cells as vehicles for CARs, we first evaluated different activation methods to facilitate both transduction and expansion to sufficient numbers for adoptive transfer. CD3/CD28 antibodies and ZOL and ConA activation of peripheral blood mononuclear cells (PBMCs) from healthy donors all led, to varying degrees, to expansion of γδT cells, as well as alpha beta T (αβT) cells. ConA and ZOL led to preferential γδT cell expansion (Figures 1A–1D). As expected, ZOL preferentially expanded the Vδ2 subtype (more than 80% purity by day 13 post-activation) (Figures 1C and 1F). In contrast, ConA led to expansion of both Vδ1 and Vδ2 cells (Figures 1D and 1G), although most cultured cells remained αβ T cells by day 13 despite significantly greater fold expansion of Vδ1 and Vδ2 cells compared to αβ (Figures 1D and 1G). There was also a high degree of inter-donor variability of fold expansion following ConA stimulation, possibly reflecting different degrees of priming of blood γδT cells in different individuals. Nevertheless, ConA was identified as a possible method for expansion of the rarer Vδ1 subset.

Bulk Populations of γδT Cells Are Efficiently Transduced with a GD2-Specific CAR and Demonstrate Antigen-Specific Cytotoxicity

Bulk populations of CD3/CD28-, ZOL-, and ConA-activated cells were efficiently transduced with a second-generation CAR targeting GD2 and containing CD3-ζ and CD28 signaling domains (GD2-CAR). CD3/CD28-activated cells were transduced with gammaretroviral supernatant 48 hr after the initial activation as previously described.[4] ConA and ZOL-activated cells were transduced 5 days post-stimulation, which had been identified as the optimal time point for maximal transduction efficiency and proliferation (Figure S1). Transduction efficiency, as determined by flow cytometry (Figure 2A), was greatest for CD3/CD28-activated αβ T cells, (mean 61.57%) (Figure 2B), although there was marked variability in transduction efficiency between donors (n = 9). Transduction following ZOL activation was highest in Vδ2 cells, which is unsurprising given that αβ and Vδ1 failed to expand in ZOL cultures (Figure 2C). Following ConA activation, there was no significant difference between transduction efficiency of different T cell subsets, with all showing 20%–40% transduced cells, somewhat lower than with CD3/CD28 stimulation (Figure 2D). Therefore, both ZOL and ConA stimulation are capable of stimulating and transducing γδT cells from peripheral blood. ^{51}Chromium (^{51}Cr) release assays were used to investigate whether γδT cells transduced with second-generation GD2-CAR exerted specific tumor cell lysis. γδT cells, in the absence of CAR, have been shown to kill a variety of tumors,[24–28] enhanced by addition of opsonizing antibody[19] or target sensitization with ZOL.[29] We have previously shown that artificial APC-expanded Vδ1 cells are capable of antibody-independent killing of certain $GD2^+$ neuroblastoma cell lines but that Vδ2 γδT cells demonstrate a lesser degree of innate killing.[19] We initially evaluated the killing properties of bulk GD2-CAR-transduced T cell populations following ZOL and ConA stimulation, using GD2-expressing LAN1 neuroblastoma cells as targets. Efficient cytotoxicity of LAN1 targets was observed using either stimulation protocol (Figures 2F and 2G), and the level of killing by γδ CAR-T cells at an effector-to-target (E:T) ratio of 10:1 was broadly equivalent to that observed with αβ CAR-T cells transduced with the same receptor following CD3/CD28 stimulation (Figure 2E). There was relatively little innate background killing by mock-transduced γδT cell controls (Figures 2F and 2G) against this particular neuroblastoma cell line. The antigen-specific nature of the cytotoxicity was confirmed by demonstration of effective killing of SupT1 cells engineered to express GD2, while there was negligible killing of SupT1 transduced with irrelevant control antigen (Figure S2).

Purified Populations of CAR-Transduced γδT Cell Subsets Retain Antigen-Specific Killing

To evaluate the relative antigen-specific killing properties of specific γδT cell subsets, bulk cell populations underwent fluorescence-activated cell sorting (FACS) to greater than 95% purity to generate populations of CAR-expressing $Vδ1^+$ and Vγ9Vδ2 subtype ($Vδ2^+$) cells, as well as control αβ CAR-T cells. Both Vδ1 ($ConA^+$) and Vδ2 ($ConA^+$ or ZOL^+) T cells transduced with GD2-CAR effectively lysed $GD2^+$ LAN1 (Figures 3A, 3B, and 3D–3F). Specific tumor cell lysis by CAR^+ γδT cells was equivalent to that observed with $CD3/CD28^+$-stimulated $GD2\text{-}CAR^+$ αβ T cells. Tumor cell killing by non-transduced (NTD) cells of all subsets was minimal. Antibody-dependent cellular cytotoxicity (ADCC) of LAN1 by NTD Vδ2, (using anti-GD2 monoclonal antibody Ch14.18/Chinese

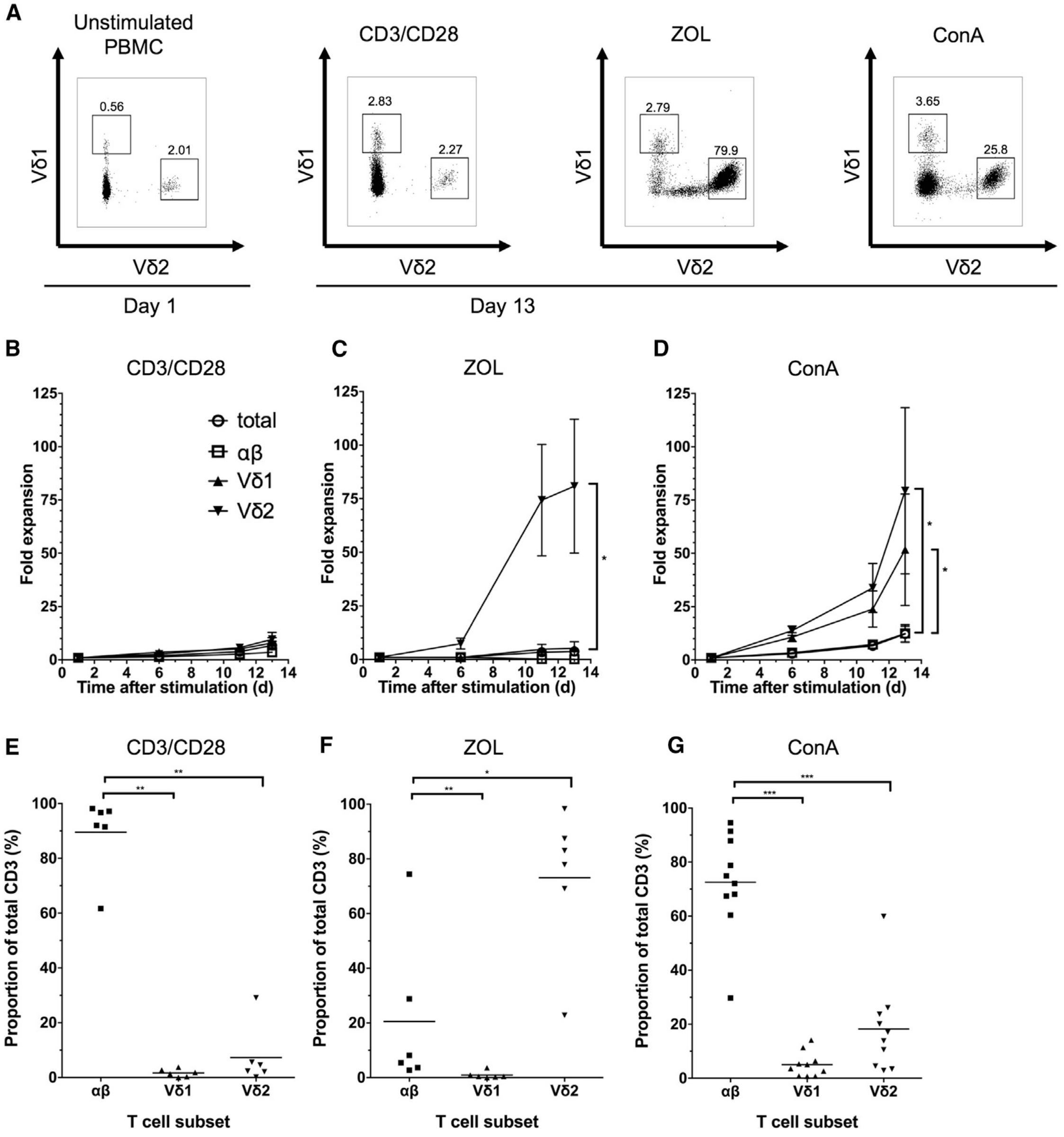

Figure 1. T Cells (αβ, Vδ1$^+$, and Vδ2$^+$) Are Successfully Expanded from Healthy Donor PBMCs Using Three Activation Methods

Cells were expanded using (1) CD3/CD28 antibody and IL-2; (2) zoledronate (ZOL) and IL-2; and (3) concanavalin A (ConA) and IL-2/IL-4. (A) Representative dot plots from a single donor showing the proportion of Vδ1$^+$ and Vδ2$^+$ cells (in a live cell gate) at baseline (left) and 13 days following activation. (B–D) αβ, Vδ1$^+$, and Vδ2$^+$ fold expansion was calculated by counting the total number of live cells by trypan blue exclusion and determining the T cell subset proportion by flow cytometry (data represented as mean ± SEM; 6 individual donors). (E–G) Preferential T cell subset expansion from PBMCs 12 days following activation with CD3/CD28 antibody (E), ZOL (F), or ConA (G). T cell subsets were determined by flow cytometry. Each data point represents an individual donor, and each horizontal line represents the mean value.

hamster ovary [CHO]) was equivalent to the killing of LAN1 by GD2-CAR$^+$ Vδ2 cells in the absence of antibody (Figure 3C). Therefore, GD2-CAR$^+$ Vδ2 T cells expanded by ZOL and polyclonal GD2-CAR$^+$ γδT cells expanded by ConA are both capable of antigen-specific killing that is at least equivalent to those of conventionally activated GD2-CAR αβ T cells, and Vδ2 T cells are additionally capable of effective ADCC.

ConA-Expanded γδ CAR-T Cells Have a Less Differentiated Phenotype and Relatively Low Expression of Exhaustion Markers

γδT cell differentiation into effector or memory cells has been described by Dieli et al.[30] Antibody staining for CD27 and CD45RA divides subsets into 4 memory phenotypes: naive memory (CD27$^+$/CD45RA$^+$) (T_{Naive}), central memory (CD27$^+$/CD45RA$^-$)

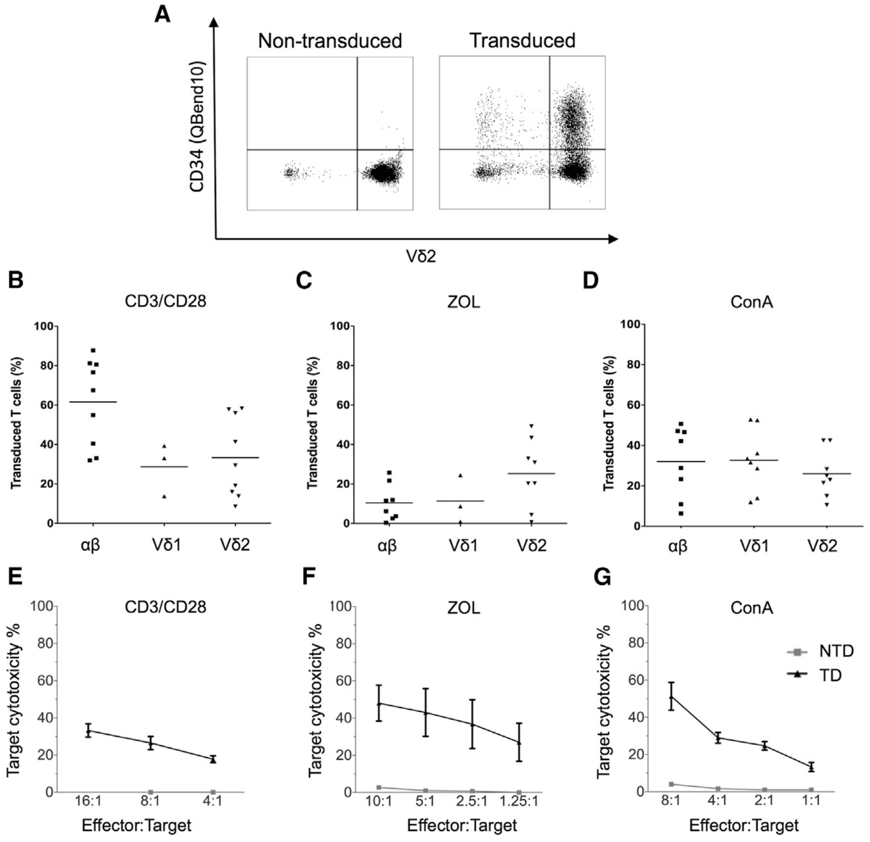

Figure 2. αβ, Vδ1$^+$, and Vδ2$^+$ T Cells Are Efficiently Transduced with GD2-CAR following Activation with CD3/CD28 Antibody, ZOL, or ConA, and Bulk Populations Are Cytotoxic to Neuroblastoma Cells

(A) Representative flow cytometry dot plot showing transduction efficiency of ZOL-expanded non-transduced and GD2-CAR$^+$-transduced PBMCs. Vδ2$^+$ populations were gated on CD3$^+$ live cells 8 days following transduction. The GD2-CAR construct coexpresses the QBend10 epitope from CD34, allowing detection by flow cytometry. Transduction efficiency was determined by the percentage of QBend10$^+$ in the T cell population gate compared to control non-transduced cells. (B–D) Mean transduction efficiency using CD3/CD28 antibody, ZOL, or ConA activation methods, respectively. Each data point represents an individual donor (n = 9) and each horizontal line is the mean. (E–G) Bulk populations of GD2-CAR-transduced T cells stimulated by CD3/CD28 antibody (E), ZOL (F), and ConA (G) specifically lyse the GD2-expressing neuroblastoma cell line, LAN1, in 4-hr ^{51}Cr release assay. NTD, non-transduced T cells; TD, transduced GD2-CAR$^+$ T cells (data represented as mean ± SEM; 3–5 individual donors in triplicate).

(T_{CM}), effector memory (CD27$^-$/CD45RA$^-$) (T_{EM}), and terminally differentiated effector memory (CD27$^-$/CD45RA$^+$) (T_{TEMRA}). T_{CM} has the highest proliferative potential and express lymph node homing receptors but has a relative lack of immediate effector function. T_{EM} is highly cytotoxic but has lower proliferative capacity.[31] αβ T cells showed a general shift from a predominantly T_{naive} and T_{CM} phenotype to the T_{CM}/T_{EM} phenotype at day 13 following activation with CD3/CD28 antibody (Figures 4A–4D). In contrast with αβ T cells, a large number of NTD and transduced Vδ1 cells (activated with ConA) maintained a CD27$^+$/CD45RA$^+$ naive phenotype, which was not affected by prolonged culture or transduction with GD2-CAR (Figures 4C and 4D). In contrast, Vδ2$^+$ cells following activation with ZOL adopted a predominantly T_{EM} phenotype, with few naive or T_{CM} cells left in culture in both the absence (Figure 4C) and the presence (Figure 4D) of CAR. The Vδ2$^+$ cells are more differentiated than Vδ1$^+$ cells and αβ T cells in unstimulated PBMCs, presumably reflecting previous activation of this circulating population through engagement of the Vγ9Vδ2 TCR. Additional stimulation of the TCR induced by ZOL further differentiates the cells, whereas neither ConA stimulation nor CAR expression significantly increase differentiation of Vδ1$^+$ γδT cells.

Programmed cell death protein 1 (PD1) and T cell immunoglobulin domain and mucin domain 3 (TIM3) are activation-induced coinhibitory receptors associated with T cell exhaustion. Despite the costimulatory effects of the CD28 endodomain contained in CAR$^+$ T cells, antigen exposure can cause upregulation of coinhibitory receptors, resulting in T cell cytotoxic dysfunction, impaired cytokine production, and high rates of apoptosis.[32,33] For αβ T and Vδ2$^+$ cells, transduction with GD2-CAR, even in the absence of cognate antigen, resulted in higher expression of TIM3 and PD1 compared to their NTD counterparts (Figures 4E and 4F). Vδ1$^+$ cells had the least exhausted phenotype, with little upregulation of TIM3 and PD1 following transduction compared to CAR$^+$ αβ T cells and Vδ2$^+$ γδT cells. Altogether, these data demonstrate that Vδ1 CAR-T cells, obtained by ConA activation and expansion, have a more naive and less exhausted phenotype than conventional GD2-CAR$^+$ αβ T cells.

Expanded γδ CAR-T Cells Display Further Antigen-Specific Proliferation and Retain Capacity to Migrate toward Tumor Cells

Antigen-specific γδ CAR-T cell proliferation was investigated by coculture of effectors (14 days after initial stimulation) with irradiated GD2$^+$ LAN1 targets (Figures 5A and 5B). First, this was evaluated in bulk-transduced lines following ConA expansion in the presence of interleukin (IL)-2. Here addition of irradiated target cells led to a mean of 3-fold further proliferation of CAR-transduced cells, although CAR alone in the absence of target also resulted in a more modest proliferation, consistent with tonic signaling from the CAR. Similar results were obtained with the different CAR$^+$ effectors (αβT, Vδ1$^+$, and Vδ2$^+$) (Figure 5A). Antigen-specific proliferation was also evaluated using ZOL-expanded Vδ2 CAR-T cells that underwent FACS in which a mean of 2.5-fold expansion was observed over 3 days in CAR$^+$ T cells in the presence of LAN1, while there was no

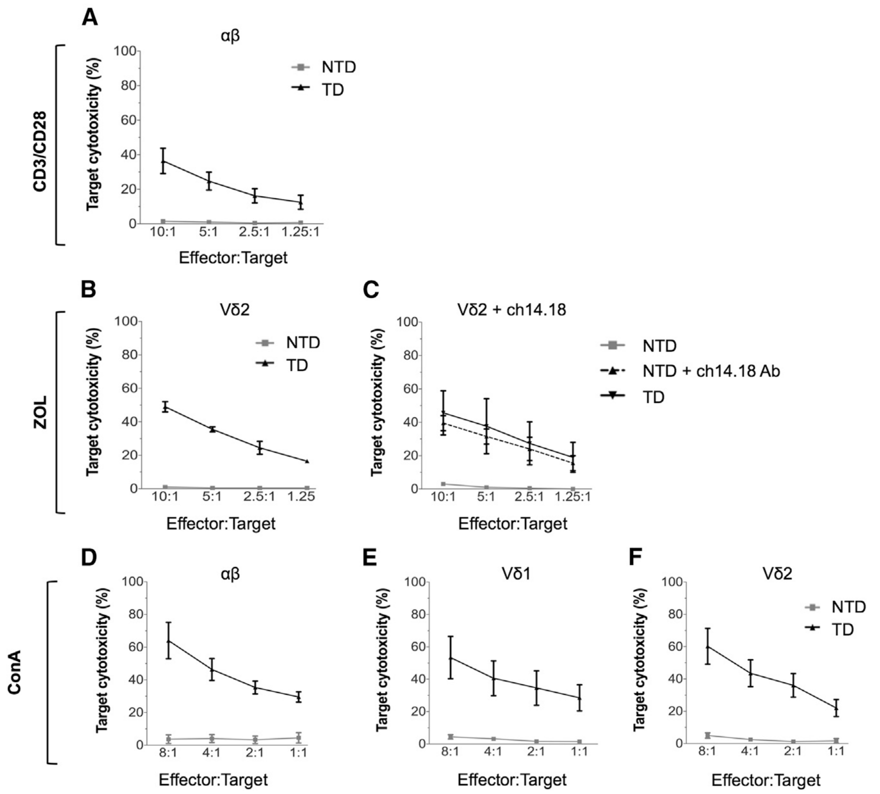

Figure 3. Purified Populations of Vδ1⁺ and Vδ2⁺ T Cells Transduced with GD2-CAR Are Each Capable of Antigen-Specific Lysis of LAN1 Neuroblastoma Cells

GD2-CAR-transduced (TD) αβ, Vδ1⁺, and Vδ2⁺ T cells were sorted to >95% purity by fluorescence-activated cell sorting (FACS). Non-transduced (NTD) cells underwent either FACS or magnetic-activated cell sorting (MACS). (A) αβ T cells were stimulated with CD3/CD28 antibody. (B and C) Vδ2⁺ cells were stimulated with ZOL, and cytotoxicity was evaluated in 4-hr ^{51}Cr release assay. In (C), ADCC of NTD cells was evaluated by adding LAN1 targets in the absence or presence of anti-GD2 antibody (Ch14.18). (D–F) ConA-activated NTD and GD2-CAR-transduced αβ, Vδ1⁺, and Vδ2⁺ CAR⁺ T cells underwent FACS to >95% purity and were cocultured with LAN1 for 4-hr ^{51}Cr release assay (data represented as mean ± SEM; 3–5 individual donors in triplicate).

significant increase in numbers in the absence of target and/or CAR (Figure 5B).

The natural tissue residency of most γδT cells, and the association of tumor infiltration by γδT cells with favorable prognosis,[9] led us to speculate that cells expanded and transduced from blood might have favorable migration toward tumor cells for adoptive cell therapy. To explore the migratory capacity of CAR⁺ γδT cells toward neuroblastoma cells, a 4-hr transwell migration assay was performed. We first evaluated background migration due to T cell motility in the absence of any cells in the lower chamber and found that between mean 5% and mean 12% of the total population traverse the wells, with no significant differences in migration properties of the CAR-transduced and NTD cells (Figure 5C). The background values were calculated for each cell type and subtracted from migration in the presence of a tumor cell stimulus to identify stimulus-specific migration (Figure 5D). It was found that all CAR⁺ cells had positive mean values for migration toward two neuroblastoma cell lines, and there were no significant differences among Vδ1⁺, Vδ2⁺ and αβ CAR-T cells (Figure 5D). Similarly, we showed for each of the subtypes that the presence of the CAR did not significantly affect migratory properties *in vitro* (Figure 5E). CAR⁺ γδT cells also migrated toward supernatant from neuroblastoma cell lines (Figure 5E), and supernatant taken from neuroblastoma neurosphere lines derived from primary patient tissue (Figure S3). Hence, γδ CAR-T cells expanded by ConA retain chemo-attraction toward 5 of 5 neuroblastoma models, consistent with a potential role for these cells in adoptive transfer immunotherapy for this disease.

Expanded and CAR-Transduced γδT Cells Acquire a pAPC Phenotype

A characteristic of professional APCs (pAPCs) is cell surface expression of costimulatory ligands such as CD86 and CD80, as well as upregulation of major histocompatibility complex (MHC) class II. Following expansion and transduction with ZOL stimulation, Vδ2⁺ cells acquired strong surface staining for CD86 and human lymphocyte antigen (HLA)-DR (Figure 6A).

Cross presentation to MHC class I molecules is a hallmark of pAPCs. To test the ability of the expanded γδ CAR-T cells to function as cross-presenting pAPCs, we used the model cancer testis antigen MART1. Responder αβ T cells were labeled with CellTrace and transduced with a recombinant αβTCR with high-affinity specificity for a known 10 amino acid MART1 peptide/HLA-A0201 complex.[34] CellTrace dilution was assessed within the MART1 αβTCR (V-beta 12 [Vβ12] chain)-positive and (Vβ12 chain)-negative gate, distinguishing antigen-specific and non-specific proliferation, respectively (Figures 6B and 6C). Using as a source of antigen for cross presentation a 25-mer MART1 long peptide (L-MART1) containing the 10-mer sequence, antigen-specific proliferation can only be detected if there is endocytosis and intracellular processing by pAPCs.

To test the ability of expanded and transduced γδ CAR⁺ T cells to function as pAPCs, we used blood from HLA-A201⁺ donors as the source of both γδ CAR⁺ T cells, as well as the autologous MART1 TCR⁺ responders. To exclude the possibility of minor populations of other APCs in the ZOL-expanded γδ CAR⁺ T cells contributing to antigen presentation, we performed FACS for the Vδ2-CAR

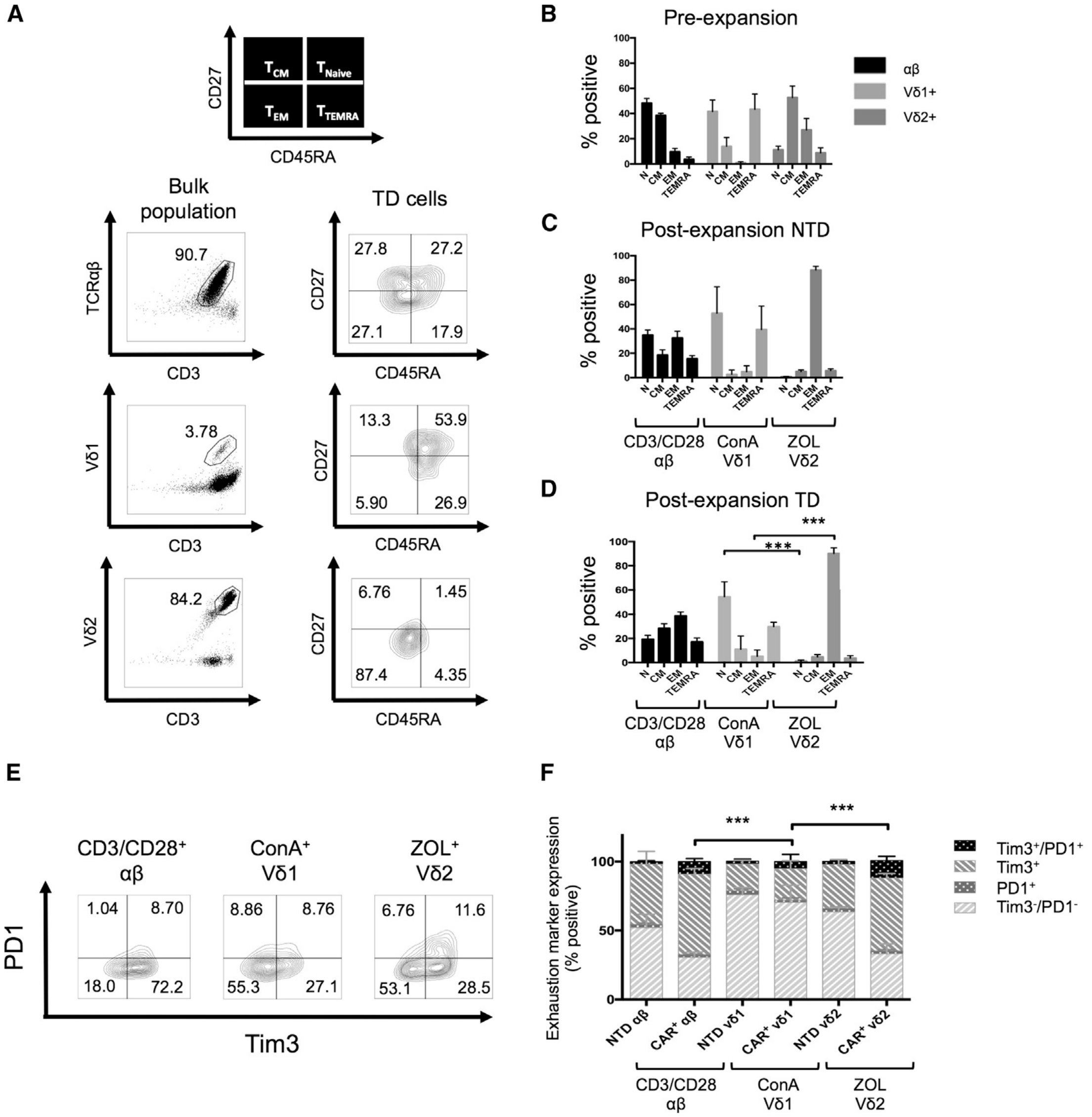

Figure 4. Memory Phenotype and Exhaustion Marker Expression on Expanded CD3+ αβ, Vδ1+, and Vδ2+ Live T Cells

(A) Representative flow cytometry dot plot showing gating of T cell subtypes (αβ, Vδ1+, and Vδ2+) and contour plots of the corresponding subtype memory phenotype (N, naive; CM, central memory; EM, effector memory; TEMRA, terminally differentiated effector memory) according to expression of CD27 and CD45RA. (B–D) memory phenotypes of pre-expanded PBMCs (B), post-expansion non-transduced (NTD) cells (C), and post-expansion GD2-CAR-transduced cells (D). αβ cells were stimulated with CD3/CD28 antibody, Vδ1 cells were stimulated with ConA, and Vδ2 cells were stimulated with ZOL (data represented as mean ± SEM; αβ and Vδ2, n = 6; Vδ1, n = 3). (E and F) Expanded Vδ1+ cells transduced with CAR after ConA stimulation express significantly fewer exhaustion markers (PD1 and TIM3) than αβ and Vδ2+ CAR-T cells. (E) Flow cytometry plots from a representative donor. αβ cells were stimulated with CD3/CD28 antibody, Vδ1+ cells were stimulated with ConA, and Vδ2+ cells were stimulated with ZOL. (F) PD1 and TIM3 expression on TD and NTD T cells 13 days following activation (data represented as mean ± SEM; αβ and Vδ2+, n = 6; Vδ1+, n = 3; p value for significance comparing double-negative populations).

population to purity with stringent gating. MART1 short peptide (S-MART1) pulsing of the γδ CAR+ T cells led to robust proliferation only of the Vβ12+ population. Long peptide pulsing also led to antigen-specific proliferation, but only in the presence of cross-presenting γδ CAR-T cells (Figure 6C). As expected, given the requirement for intracellular processing, long peptide-specific proliferation was less robust than short peptide-pulsed cells. The ability to cross present was found in γδ CAR-T cells from a range of donors (Figure 6D).

DISCUSSION

Engineering of CAR-T cell therapy requires a fine balance between maximizing efficacy of tumor killing and reducing treatment-related morbidity, including on-target, off-tumor effects. Successful

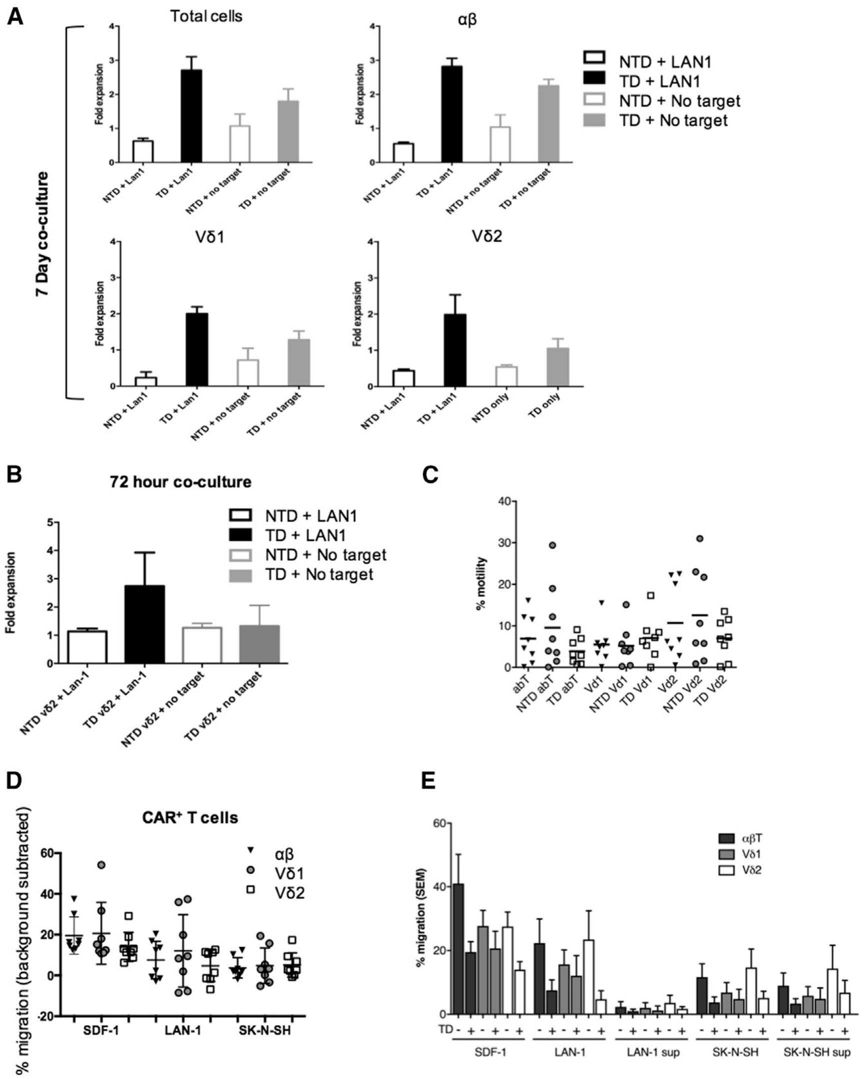

Figure 5. CAR+-Transduced γδT Cells Exhibit Enhanced Proliferation and Migrate toward Neuroblastoma Cells *In Vitro*

(A) Bulk ConA-expanded T cells were cocultured with irradiated LAN1 cells at an E:T ratio of 1:1 for 7 days in the presence of IL-2. Fold expansion was calculated by counting the total number of live cells by trypan blue exclusion and then determining the T cell subset proportion by flow cytometry. (B) ZOL-expanded TD Vδ2+ and NTD Vδ2+ underwent FACS and were cocultured with irradiated LAN1 cells for 72 hr in the absence of IL-2. Fold expansion was calculated by trypan blue exclusion (data represented as mean ± SEM; n = 4). (C) Percentage T cell motility from the upper to the lower chamber in the absence of any stimulus in the lower chamber in a 4-hr transwell migration assay. (D) Analysis of CAR cells of αβ, Vδ1+, and Vδ2+ subtypes migrating toward GD2-expressing LAN1 and GD2− SK-N-SH neuroblastoma cells in 4-hr transwell migration assay. SDF-1 was used as a positive chemokine control. Scatter dot plots (line at mean) represent the percentage of migration (n = 8 donors). (E) Percentage T cell migration toward SDF-1 (positive control), neuroblastoma cell lines (LAN1 and SK-N-SH), and neuroblastoma cell line supernatant (LAN1 sup and SK-N-SH sup) in 4-hr transwell migration assay. Background motility for each cell type was subtracted from migration in the presence of stimulus to identify stimulus-dependent percentage migration. The bulk population used in the chemotaxis assay contained a mixture of αβ T cells (dark gray bars), Vδ1+ T cells (light gray bars), and Vδ2+ T cells (white bars). Comparison was made between transduced (TD+) and non-transduced (TD−) T cells. Data show mean values (+SEM) of eight donors. In (D) and (E), percentage of migration calculated as (number of migrated cells in the specific condition − number of migrated cells in the negative control for that condition) / number of migrated cells in the positive control) × 100.

treatment of solid tumors also depends on the ability of CAR-T cells to home to the tumor site, overcome the immunosuppressive tumor microenvironment, and persist long term. Conventional approaches use αβ CAR-T cells, which have resulted in impressive clinical responses in leukemia but have yet to be translated to equivalent successes in solid cancers. We identified γδ CAR-T cells as a potential novel approach due to their additional desirable functional properties. Specifically, γδ CAR-T cells, as a first line of defense, are already primed for innate cytotoxicity;[8] have natural residence in the solid tumor microenvironment;[9] and have been shown following activation to acquire phenotype and properties of profession APCs.[13–16] We therefore tested the hypothesis that following transduction with a CAR, γδ CAR-T cell products would be obtained and that these would retain the innate anti-cancer properties, as well as acquiring additional antigen-specific cytotoxicity and proliferative response. As a first proof of concept, we have used a second-generation CD28ζ endodomain-containing CAR and GD2 as a target antigen suitable for many solid cancers, including neuroblastoma. While further optimization is necessary, the data demonstrate retention of the important γδT functions of antigen cross presentation to effector T cells and migration toward tumor cells while gaining additional antigen-specific cytotoxicity.

γδ CAR-T cells were expanded from the PBMCs of healthy donors using three T cell stimulation methods: CD3/CD28 antibody, ZOL, and ConA, of which CD3/CD28 antibody and ZOL are already available in good manufacturing practice (GMP) form. ZOL *ex vivo* expansion of Vγ9Vδ2 cells for adoptive transfer was also previously described[35] and used in early clinical trials.

ConA expansion for clinical applications would require further development but is an attractive proposition because of its more polyclonal expansion of γδT cells, including the more naive Vδ1+ subset, and an increased frequency of cells lacking PD1 and TIM3 expression

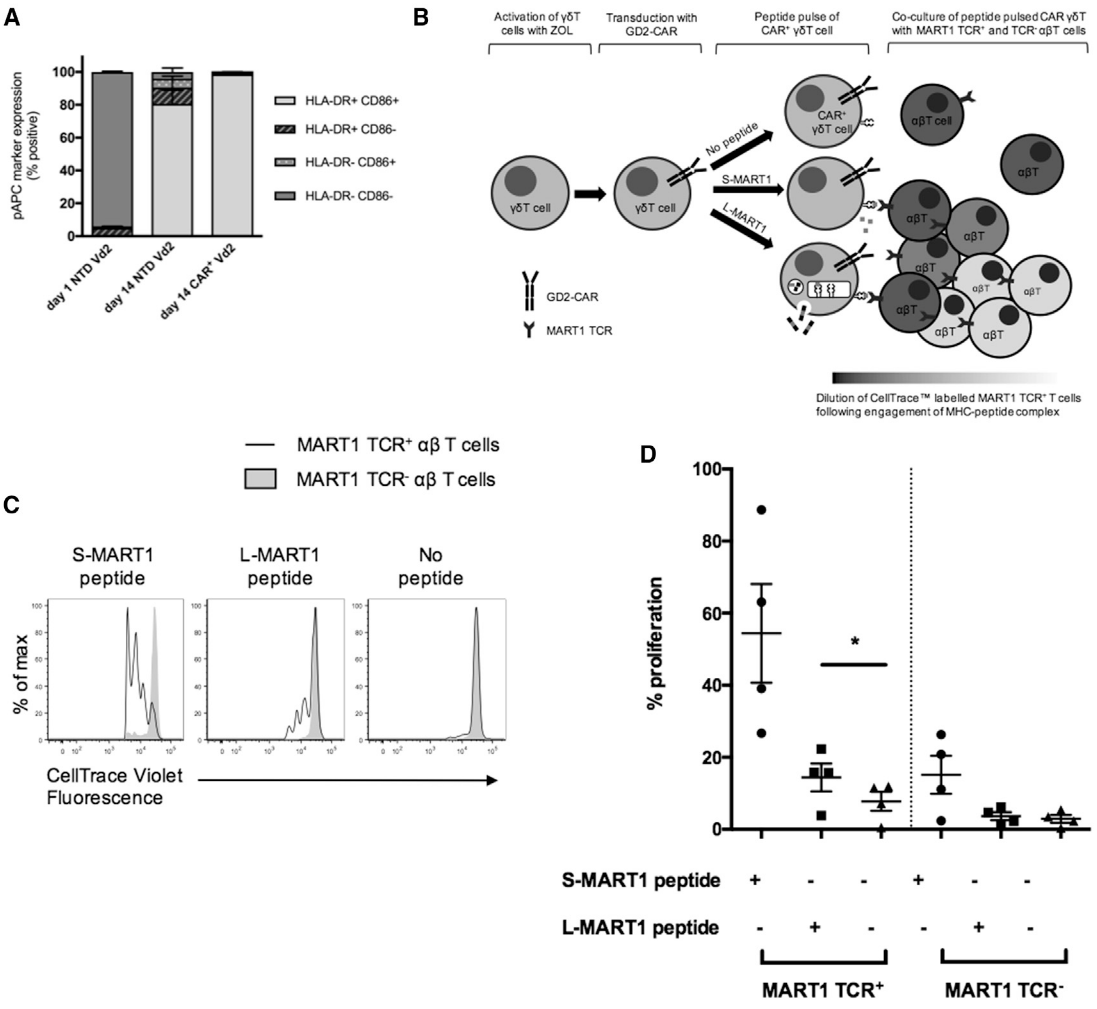

Figure 6. CAR$^+$ Vδ2$^+$ T Cells Can Cross Present MART1 Tumor Antigen to Responder $\alpha\beta$ T Cells Transduced with a Cell-Transduced $\alpha\beta$TCR

(A) Expression of CD86 and HLA-DR on pre-expanded Vδ2$^+$, post-expanded non-transduced (NTD) Vδ2$^+$, and CAR$^+$ Vδ2$^+$ from multiple donors (data represented as mean ± SEM; n = 3). (B) Schematic representation of a GD2-CAR$^+$ Vδ2$^+$ T cell functioning as a professional antigen-presenting cell by endocytosis and intracellular processing of L-MART1 protein. Following antigenic stimulation, CellTrace violet-labeled MART1 TCR$^+$ $\alpha\beta$ T cells proliferate, resulting in dilution of violet dye. (C) Representative histogram showing proliferation as determined by CellTrace violet dilution. (D) Proliferation of responder $\alpha\beta$ T cells (MART1 TCR$^+$ and MART1 TCR$^-$) in multiple donors (horizontal line represents mean value; paired Student's t test; n = 4). Higher proliferation of Vβ12$^-$ cells in S-MART1 conditions is probably due to bystander activation by the Vβ12$^+$ responders. The small background proliferation in Vβ12$^+$ cells in the absence of peptide is commonly seen following transduction.

compared with the other methods; in addition, Vδ1$^+$ cells have been shown to have enhanced tumor killing, tissue penetration, and resistance to activation-induced cell death compared with Vδ2$^+$ cells.[18,36] Greater numbers of CAR$^+$ Vδ1$^+$ are potentially achievable through, for example, enrichment by cell separation techniques during production, as described by Almeida et al.,[22] or culturing in the presence of IL-2 and IL-7, as previously described.[37] ConA expansion might be of particular interest in childhood cancers, because Vδ1$^+$ cells are known to predominate during fetal development and childhood but by adulthood, most $\gamma\delta$T cells in peripheral blood are Vγ9Vδ2 cells.[38]

Vδ1$^+$ and Vδ2$^+$ $\gamma\delta$T cell subsets were successfully transduced with GD2-CAR using a gamma-retroviral vector following ZOL or ConA stimulation with adequate transduction efficiency for adoptive transfer, albeit somewhat lower than that observed with CD3/CD28-activated cells. This difference is probably explainable by the gentler stimulus of ZOL or ConA and could be ameliorated by using methods of gene transfer less dependent on cell division, such as lentiviral transduction or electroporation.

We have previously shown that expanded Vδ1$^+$ cells retain innate killing of neuroblastoma cells,[19] while Vδ2$^+$ cells lose cytotoxicity during expansion.[16,19] In the current study, the innate cytotoxicity of the expanded cells following CD56 depletion was negligible against neuroblastoma (Vδ1 and Vδ2) (Figure 2) and low against SupT1 cells (Vδ2) (Figure S2). Although further studies will be required to

determine how much innate killing activity is preserved in expanded and transduced cells, the important message of our study is that CAR-dependent cytotoxicity is equivalent to that seen with conventional CAR αβ T cells. The γδ CAR-T cells are capable of further expansion following a second stimulation augmented by the presence of the cognate antigen for the CAR. A second-generation GD2-CD28-CD3-ζ CAR was chosen for this study because of the capacity of CD28 CARs for rapid acute response.[39] Future studies might assess tumor necrosis factor (TNF) receptor superfamily CAR endodomains for their capacity for more sustained expansion.[32,39,40]

Most significantly, we have shown for the first time that expanded and CAR-transduced γδT cells are capable of migrating toward tumor cells and of antigen cross presentation. This finding opens a new avenue of research, because it suggests that a CAR-T cellular product has the capacity to enter the tumor site, where it is responsible for both killing of tumor cells and release of tumor-associated antigens, as well as uptake of released antigens, leading to stimulatory antigen presentation to tumor-infiltrating lymphocytes. This approach is likely to be of interest in diseases like melanoma with high tumor antigen frequency and large numbers of tumor-reactive, tumor-infiltrating lymphocytes. We conclude that γδ CAR-T cells can be generated in sufficient number for adoptive transfer immunotherapy for cancer and have potent tumor antigen-dependent cytotoxicity. Their capacity for migration and for uptake and cross presentation of tumor-associated antigens marks them as having potential advantages over conventional CAR-T cells, especially in the solid tumor setting.

MATERIALS AND METHODS

Cell Lines and Primary Cells

Human neuroblastoma cell lines LAN1 and SK-N-SH and a lymphoblastic lymphoma cell line (SupT1) were obtained from the ATCC. Fresh blood samples were obtained from healthy laboratory donors in accordance with protocols approved by the UK Integrated Research Ethics Review, after obtaining informed consent.

Activation and Expansion of T Cells

PBMCs were isolated from whole blood by density gradient centrifugation (Lymphoprep, Stem Cell Technologies, Cambridge, UK). Two methods for γδT cell expansion were employed. First, for selective expansion of the Vγ9Vδ2 subtype (Vδ2$^+$), PBMCs were resuspended in medium (RPMI, 10% fetal calf serum [FCS], 1% penicillin/streptomycin [PS]) supplemented with 100 IU/mL IL-2 (Proleukin, Prometheus, Switzerland) and 1 μg/mL ZOL (Zerlinda at 4 mg/100 mL, Actavis). Second, for the expansion of polyclonal γδT cells, including those bearing the Vδ1$^+$ and Vδ2$^+$ TCR, PBMCs were resuspended in medium (RPMI, 10% FCS, 1% PS) supplemented with 100 IU/mL IL-2, 10 ng/mL recombinant IL-4 (Cellgenix), and 1 μg/mL ConA (Sigma-Aldrich). αβ T cells were activated and expanded using 1 μg/mL soluble anti-CD3 (clone OKT3, Miltenyi) and anti-CD28 antibodies (clone 15E8, Miltenyi) (CD3/CD28). All cell cultures were incubated at 37°C and 5% CO_2 and maintained at a cell density of 1×10^6/mL. Fresh media containing 100 IU/mL IL-2 (and 10 ng/mL IL-4 for ConA-activated cells) was replenished every 2–3 days.

CAR

The clinical-grade retroviral vector SFGmR.RQR8-2A-aGD2-huK666-HCH2CH3pvaa-CD28Z (referred to as GD2-CAR hereafter) was used for all studies. The construct includes two transgenes: a second-generation CAR comprising the ScFv from murine anti-GD2 antibody (muK666),[41] which has subsequently been codon optimized and humanized to form huK666.[42] huK666 is fused with an immunoglobulin G (IgG) Fc spacer, a CD28 transmembrane domain, and the CD28 and CD3-ζ intracellular signaling domains. The marker/suicide gene, RQR8, is coexpressed with the CAR using a foot and mouth virus, self-cleaving 2A sequence as previously described.[43] Coexpression of RQR8 allows elimination of CAR$^+$ T cells by anti-CD20 monoclonal antibody (Rituximab) should toxicities occur, and the target epitope from CD34, which is targeted by the anti-CD34 clone QBend10, allows detection of transduced GD2-CAR$^+$ cells by flow cytometry.

Production of Retroviral Supernatant and Transduction of T Cells

High-titer retroviral supernatant pseudotyped with the RD114 envelope was generated by incubating infected HEK293 cells for 48 hr in culture medium (90% DMEM, 10% FCS, 4 mM L-glutamine, 1% PS) at 37°C and 5% CO_2. Following incubation, supernatants were frozen and stored at −80°C until further use.

For production of γδ CAR$^+$ T cells, PBMCs stimulated with ZOL (+IL-2 at 100 IU/mL) or ConA (+IL-2 at 100 IU/mL and IL-4 at 10 ng/mL) were transduced in 24-well plates pre-coated with recombinant fibronectin fragment (RetroNectin, Takara, Japan), 5 days following initial activation. 48 hr after transduction, γδ CAR-T cells were expanded with IL-2 (100 IU/mL) added every 2–3 days. For αβ T cell control experiments, CD3/CD28 antibody-stimulated PBMCs were transduced 48 hr following the initial activation and thereafter treated according to the same protocol as for γδ CAR-T cells.

Flow Cytometry

All flow cytometry data were acquired on BD LSRII flow cytometer, and results were analyzed using FlowJo software (v.X.0.7, Tree Star, Ashland, OR). Compensation was carried out using single-color controls of either cells or beads (OneComp eBeads, eBioscience). Appropriate isotype controls, fluorescence minus one (FMO), or NTD cells were used to validate gating. All samples were stained with a LIVE/DEAD Fixable Aqua Dead Cell Stain Kit (Life Technologies) or Fixable Viability Dye eFluor 780 (eBioscience) before antibody staining. The following fluorochrome-conjugated mouse anti-human antibodies were used: CD3 PerCP-Cy5.5 (BioLegend, clone UCHT1), Vδ2 PE (BioLegend, clone B6), Vδ2 fluorescein isothiocyanate (FITC) (Miltenyi, clone 123R3), Vδ1 APC-Vio770 (Miltenyi, clone REA173), Vδ1 PE (Miltenyi, clone REA173), anti-TCR γδ PE-Vio770 (Miltenyi, clone 11F2), anti-TCR αβ Brilliant Violet (BV) 421 or PE-Cy7 (both BioLegend, clone IP26), QBend10 APC (R&D Systems, clone 4H11), CD27 BV711 (BioLegend, clone O323), CD45RA PE-Cy7 (BioLegend, clone H100), PD1 FITC (BioLegend,

clone EH12.2H7), TIM3 BV605 (BioLegend, clone F38-2E2), and TCR Vβ12 FITC (Abcam, clone S511).

Isolation of Pure T Cell Populations

Thirteen days following initial activation, transduced T cells were sorted into pure populations of GD2-CAR$^+$ αβ, Vδ1$^+$, and Vδ2$^+$ by FACS using a BD FACSAria flow sorter. ZOL-activated cells were stained with Vδ2 PE antibody and QBend10 APC. Vδ2$^+$/QBend10$^+$ cells were selected for further analysis. Transduced CD3/CD28 antibody-activated cells were stained with CD56 PE (BioLegend, clone 188) and QBend10 APC antibodies; CD56$^-$/QBend10$^+$ cells were selected for further analysis.

NTD control CD3/CD28 antibody-activated αβ cells were depleted of CD56$^+$ natural killer (NK) cells by CD56 MicroBeads according to the manufacturer's instructions (Miltenyi, 130-050-401). NTD ZOL-activated γδT cells were isolated using the TCR γ/δ+ T Cell Isolation Kit (Miltenyi, 130-092-892).

For ConA expansions, γδT cells were positively selected using TCR γ/δ MicroBeads (Miltenyi, 130-050-701) before FACS to reduce the numbers to be sorted. Pure populations of transduced (QBend10$^+$) and NTD (QBend10$^-$) αβ, Vδ1$^+$, and Vδ2$^+$ cells were obtained by staining with Vδ1 PE, Vδ2 FITC, anti-TCR γδ PE-Vio770, and QBend10 APC before sorting the respective cell populations.

Cytotoxicity Assays

In vitro cytotoxicity was assessed using standard 4-hr ^{51}Cr release assay as previously described.[19] Expanded transduced and NTD γδ and αβ cells were used as effectors, and human neuroblastoma LAN1 cells were used as targets. LAN1 cells were labeled with 100 μCi $Na_2{}^{51}CrO_4$ and cocultured with effector cells at a range of E:T ratios. Human IgG1 anti-GD2 antibody (Ch14.18/CHO) was used to opsonize GD2$^+$ LAN1 cells before labeling where stated.

Secondary Re-expansion

Day 14 ConA-expanded effector cells were cocultured with irradiated (80 Gy) LAN1 cells at a 1:1 ratio for 7 days. 1 × 10^6 effectors and 1 × 10^6 LAN1 were cocultured in 24-well plates in the presence of IL-2 (100 IU/mL). Cell density was maintained at 1 × 10^6 cells/mL, and fresh media and IL-2 were replenished every 2–3 days. After 7 days, cells were harvested and counted using trypan blue exclusion. Specific T cell subset expansion within bulk effector cultures was determined by staining for Vδ1$^+$, Vδ2$^+$, anti-TCR γδ, anti-TCR αβ, and QBend10. For ZOL-activated effectors, day 13-expanded cells were flow sorted for Vδ2$^+$ and QBend10$^+$. NTD control Vδ2$^+$ cells were purified by positive selection using anti-TCRγ/δ MicroBeads. Effectors and targets were cocultured at a 1:1 ratio for 72 hr in the absence of additional cytokines.

Cell Migration Assay

Following ConA expansion, we compared migration of Vδ1$^+$ CAR-T cells and Vδ2$^+$ CAR-T cells from 8 independent donors. Each expanded cell population contained a mixture of Vδ1$^+$, Vδ2$^+$, and αβ CAR-T cells. The migratory potential of these NTD cell populations was determined by a chemotaxis assay using 24-well culture plates carrying polycarbonate membrane-coated transwell permeable inserts (5 μm pore size; Costar Transwell, Corning, NY). 0.5 × 10^6 cells were seeded in the upper wells, and lower wells contained (1) 600 μL of supernatant of confluent LAN1 or SK-N-SH cultures, (2) 600 μL of supernatant of neuroblastoma primary cell cultures, or (3) LAN1 or SK-N-SH overnight cultures of 0.5 × 10^6 tumor cells. RPMI + 10% FCS in the lower compartment served as a negative control, representing the random background migration of immune cells, and by transferring all cells (0.5 × 10^6) to the lower well, we determined the maximum possible yield (positive control). As an assay control, we included the condition whereby the lower well contained 100 ng/mL SDF-1 (CXCL12, Sigma-Aldrich). Cell migration was allowed for 4 hr at 37°C and 5% CO_2, whereupon migrated cells were collected from the lower compartment. Counting beads (Precision Count Beads, BioLegend) were added, and then cells were resuspended in a fixed volume and counted flow cytometrically. Migration was expressed using the following equation: percentage of migration = (number of migrated cells in the specific condition − number of migrated cells in the negative control) / number of migrated cells in the positive control) × 100. We gated on both the CAR$^+$ and the NTD of each subtype within the bulk migrated population to determine whether the presence of the CAR influenced migration.

Tumor Antigen Cross-Presentation Assays

ZOL-activated, CAR-transduced Vδ2$^+$ T cells were cultured for 13 days in the presence of 100 IU IL-2 before stringent purification by FACS. After 24 hr, purified CAR$^+$ Vδ2$^+$ T cells were pulsed with cancer testis antigen MART1—short peptide (MART1$_{26–35}$, ELAGI GILTV), long peptide (MART1$_{16–40}$, GHGHSYTTAEELAGIGILT VILGVL) (ProteoGenix, France), or no peptide—for 4 hr at 37°C in serum-free medium. All peptides were used at a concentration of 5 μ/mL. Cells were then washed twice before coculture with responder αβ T cells.

Specific MART1 TCR$^+$ αβ T cells were produced from frozen autologous PBMCs taken from the same blood draw as that used for the Vδ2$^+$ T cell expansion. PBMCs were thawed on day 5 and activated with 1 μg/mL CD3, 1 μg/mL CD28 antibody, and 100 IU IL-2. 48 hr later, cells were transduced on RetroNectin-coated, 24-well plates with viral supernatant containing a recombinant αβTCR with high-affinity specificity for a known 10 amino acid MART1 peptide/HLA-A0201 complex[34] (MART1-TCR) using the identical protocol described earlier for the preparation of GD2-CAR αβ T cells.

MART1 αβTCR viral supernatant was produced by transient cotransfection of HEK293T cells with RD114 envelope protein, gag-pol, and plasmid-encoding MART1-TCR (gift from C. Cohen). In brief, 1.5 × 10^6 HEK293T cells were cultured in DMEM/10% FCS in a 100-mm dish (Nunclon Delta Surface, Thermo Fisher) for 24 hr before cotransfection using GeneJuice Transfection Reagent (Novagen/Millipore, Massachusetts, USA) according to manufacturer instructions.

Viral supernatant was harvested 48 and 72 hr after transfection and then pooled and stored at -80°C until use.

Following transduction, $\alpha\beta$ T cells were cultured for 9 days with 100 IU of IL-2 before labeling with CellTrace (CellTrace Violet Cell Proliferation Kit, Invitrogen) according to manufacturer protocol. On day 14, peptide-pulsed or control CAR$^+$ Vδ2$^+$ T cells were cocultured with MART1 TCR$^+$-transduced, CellTrace-labeled $\alpha\beta$ T cells at a ratio of 1:3 for 5 days. Proliferation of responder MART1 TCR$^+$-transduced T cells was measured flow cytometrically with CellTrace by gating on MART1 $\alpha\beta$TCR (Vβ12 chain-FITC)$^+$ cells.

Statistical Analysis

Data was analyzed using Microsoft Excel 2011 (v.14.4.5) and GraphPad Prism (v.6.0d). Unless stated otherwise, data are expressed as mean ± SEM. A two-way ANOVA with donor matching and Bonferroni post-test analysis was used to assess significance unless stated otherwise. A p value < 0.05 indicates significance (***$p < 0.001$, **$p < 0.005$, *$p < 0.05$; NS, not significant, $p > 0.05$).

SUPPLEMENTAL INFORMATION

Supplemental Information includes three figures and can be found with this article online at https://doi.org/10.1016/j.ymthe.2017.12.001.

AUTHOR CONTRIBUTIONS

A.C. and H.H.V.A. designed, performed, and analyzed experiments. G.B. performed experiments. A.M.K. and Z.A. optimized antigen cross-presentation methods. R.W., T.G., and B.F. assisted with chromium release assays. J.F., Y.M., K.G., and B.F. provided immunological support. J.A. designed experiments, provided supervision, and contributed to manuscript preparation in collaboration with A.C. and H.H.V.A.

CONFLICTS OF INTEREST

J.A. has financial interest in Autolus. J.A. and J.F. have licensed intellectual property related to $\gamma\delta$ CAR-T cells to TC Biopharm.

ACKNOWLEDGMENTS

The authors thank Dr. Martin Pule at the UCL Cancer Institute and Dr. Karin Straathof at the UCL Institute of Child Health for their technical assistance with chimeric antigen receptor design. A.C. is a clinical training fellow supported by grants from the Wellcome Trust, Great Ormond Street Hospital Children's Charity (GOSHCC), and NIHR Great Ormond Street Hospital Biomedical Research Centre. Further grant support was from Action Medical Research + GOSHCC (GN2400), Children with Cancer UK (15-175), GOSHCC infrastructure award (W1076), and Research in Childhood Cancer (RICC). H.H.V.A. holds a PhD fellowship from the Research Foundation Flanders (FWO) and received a travel grant for a long stay abroad from the FWO. J.A. is funded by a GOSHCC leadership award and the NIHR Great Ormond Street Hospital Biomedical Research Centre. J.F. is funded by a Wellcome Trust clinical fellowship (110022/Z/15/Z). The authors are also grateful to Dr. Ayad Eddaoudi and Stephanie Canning in the UCL Institute of Child Health Flow Cytometry Core Facility.

REFERENCES

1. Sadelain, M., Brentjens, R., and Rivière, I. (2013). The basic principles of chimeric antigen receptor design. Cancer Discov. *3*, 388–398.
2. Maude, S.L., Frey, N., Shaw, P.A., Aplenc, R., Barrett, D.M., Bunin, N.J., Chew, A., Gonzalez, V.E., Zheng, Z., Lacey, S.F., et al. (2014). Chimeric antigen receptor T cells for sustained remissions in leukemia. N. Engl. J. Med. *371*, 1507–1517.
3. Fousek, K., and Ahmed, N. (2015). The evolution of T-cell therapies for solid malignancies. Clin. Cancer Res. *21*, 3384–3392.
4. Pule, M.A., Savoldo, B., Myers, G.D., Rossig, C., Russell, H.V., Dotti, G., Huls, M.H., Liu, E., Gee, A.P., Mei, Z., et al. (2008). Virus-specific T cells engineered to coexpress tumor-specific receptors: persistence and antitumor activity in individuals with neuroblastoma. Nat. Med. *14*, 1264–1270.
5. Louis, C.U., Savoldo, B., Dotti, G., Pule, M., Yvon, E., Myers, G.D., Rossig, C., Russell, H.V., Diouf, O., Liu, E., et al. (2011). Antitumor activity and long-term fate of chimeric antigen receptor-positive T cells in patients with neuroblastoma. Blood *118*, 6050–6056.
6. Pulè, M.A., Straathof, K.C., Dotti, G., Heslop, H.E., Rooney, C.M., and Brenner, M.K. (2005). A chimeric T cell antigen receptor that augments cytokine release and supports clonal expansion of primary human T cells. Mol. Ther. *12*, 933–941.
7. Heczey, A., Liu, D., Tian, G., Courtney, A.N., Wei, J., Marinova, E., Gao, X., Guo, L., Yvon, E., Hicks, J., et al. (2014). Invariant NKT cells with chimeric antigen receptor provide a novel platform for safe and effective cancer immunotherapy. Blood *124*, 2824–2833.
8. Vantourout, P., and Hayday, A. (2013). Six-of-the-best: unique contributions of $\gamma\delta$ T cells to immunology. Nat. Rev. Immunol. *13*, 88–100.
9. Gentles, A.J., Newman, A.M., Liu, C.L., Bratman, S.V., Feng, W., Kim, D., Nair, V.S., Xu, Y., Khuong, A., Hoang, C.D., et al. (2015). The prognostic landscape of genes and infiltrating immune cells across human cancers. Nat. Med. *21*, 938–945.
10. Fisher, J.P., Heuijerjans, J., Yan, M., Gustafsson, K., and Anderson, J. (2014). $\gamma\delta$ T cells for cancer immunotherapy: a systematic review of clinical trials. OncoImmunology *3*, e27572.
11. Chiplunkar, S., Dhar, S., Wesch, D., and Kabelitz, D. (2009). Gammadelta T cells in cancer immunotherapy: current status and future prospects. Immunotherapy *1*, 663–678.
12. Gomes, A.Q., Martins, D.S., and Silva-Santos, B. (2010). Targeting $\gamma\delta$ T lymphocytes for cancer immunotherapy: from novel mechanistic insight to clinical application. Cancer Res. *70*, 10024–10027.
13. Khan, M.W., Curbishley, S.M., Chen, H.C., Thomas, A.D., Pircher, H., Mavilio, D., Steven, N.M., Eberl, M., and Moser, B. (2014). Expanded human blood-derived $\gamma\delta$T cells display potent antigen-presentation functions. Front. Immunol. *5*, 344.
14. Himoudi, N., Morgenstern, D.A., Yan, M., Vernay, B., Saraiva, L., Wu, Y., Cohen, C.J., Gustafsson, K., and Anderson, J. (2012). Human $\gamma\delta$ T lymphocytes are licensed for professional antigen presentation by interaction with opsonized target cells. J. Immunol. *188*, 1708–1716.
15. Brandes, M., Willimann, K., Bioley, G., Lévy, N., Eberl, M., Luo, M., Tampé, R., Lévy, F., Romero, P., and Moser, B. (2009). Cross-presenting human gammadelta T cells induce robust CD8+ alphabeta T cell responses. Proc. Natl. Acad. Sci. USA *106*, 2307–2312.
16. Barisa, M., Kramer, A.M., Majani, Y., Moulding, D., Saraiva, L., Bajaj-Elliott, M., Anderson, J., and Gustafsson, K. (2017). *E. coli* promotes human Vγ9Vδ2 T cell transition from cytokine-producing bactericidal effectors to professional phagocytic killers in a TCR-dependent manner. Sci. Rep. *7*, 2805.
17. Bonneville, M., O'Brien, R.L., and Born, W.K. (2010). Gammadelta T cell effector functions: a blend of innate programming and acquired plasticity. Nat. Rev. Immunol. *10*, 467–478.
18. Siegers, G.M., and Lamb, L.S., Jr. (2014). Cytotoxic and regulatory properties of circulating Vδ1+ $\gamma\delta$ T cells: a new player on the cell therapy field? Mol. Ther. *22*, 1416–1422.

19. Fisher, J.P., Yan, M., Heuijerjans, J., Carter, L., Abolhassani, A., Frosch, J., Wallace, R., Flutter, B., Capsomidis, A., Hubank, M., et al. (2014). Neuroblastoma killing properties of Vδ2 and Vδ2-negative γδT cells following expansion by artificial antigen-presenting cells. Clin. Cancer Res. *20*, 5720–5732.

20. Deniger, D.C., Switzer, K., Mi, T., Maiti, S., Hurton, L., Singh, H., Huls, H., Olivares, S., Lee, D.A., Champlin, R.E., and Cooper, L.J. (2013). Bispecific T-cells expressing polyclonal repertoire of endogenous γδ T-cell receptors and introduced CD19-specific chimeric antigen receptor. Mol. Ther. *21*, 638–647.

21. Siegers, G.M., Dhamko, H., Wang, X.H., Mathieson, A.M., Kosaka, Y., Felizardo, T.C., Medin, J.A., Tohda, S., Schueler, J., Fisch, P., and Keating, A. (2011). Human Vδ1 γδ T cells expanded from peripheral blood exhibit specific cytotoxicity against B-cell chronic lymphocytic leukemia-derived cells. Cytotherapy *13*, 753–764.

22. Almeida, A.R., Correia, D.V., Fernandes-Platzgummer, A., da Silva, C.L., da Silva, M.G., Anjos, D.R., and Silva-Santos, B. (2016). Delta one T cells for immunotherapy of chronic lymphocytic leukemia: clinical-grade expansion/differentiation and preclinical proof of concept. Clin. Cancer Res. *22*, 5795–5804.

23. Rischer, M., Pscherer, S., Duwe, S., Vormoor, J., Jürgens, H., and Rossig, C. (2004). Human gammadelta T cells as mediators of chimaeric-receptor redirected anti-tumour immunity. Br. J. Haematol. *126*, 583–592.

24. Kabelitz, D., Wesch, D., Pitters, E., and Zöller, M. (2004). Characterization of tumor reactivity of human V gamma 9V delta 2 gamma delta T cells in vitro and in SCID mice in vivo. J. Immunol. *173*, 6767–6776.

25. Bonneville, M., and Scotet, E. (2006). Human Vgamma9Vdelta2 T cells: promising new leads for immunotherapy of infections and tumors. Curr. Opin. Immunol. *18*, 539–546.

26. Dieli, F., Vermijlen, D., Fulfaro, F., Caccamo, N., Meraviglia, S., Cicero, G., Roberts, A., Buccheri, S., D'Asaro, M., Gebbia, N., et al. (2007). Targeting human gammadelta T cells with zoledronate and interleukin-2 for immunotherapy of hormone-refractory prostate cancer. Cancer Res. *67*, 7450–7457.

27. Bennouna, J., Bompas, E., Neidhardt, E.M., Rolland, F., Philip, I., Galéa, C., Salot, S., Saiagh, S., Audrain, M., Rimbert, M., et al. (2008). Phase-I study of Innacell gammadelta, an autologous cell-therapy product highly enriched in gamma9delta2 T lymphocytes, in combination with IL-2, in patients with metastatic renal cell carcinoma. Cancer Immunol. Immunother. *57*, 1599–1609.

28. Abe, Y., Muto, M., Nieda, M., Nakagawa, Y., Nicol, A., Kaneko, T., Goto, S., Yokokawa, K., and Suzuki, K. (2009). Clinical and immunological evaluation of zoledronate-activated Vgamma9gammadelta T-cell-based immunotherapy for patients with multiple myeloma. Exp. Hematol. *37*, 956–968.

29. Di Carlo, E., Bocca, P., Emionite, L., Cilli, M., Cipollone, G., Morandi, F., Raffaghello, L., Pistoia, V., and Prigione, I. (2013). Mechanisms of the antitumor activity of human Vγ9Vδ2 T cells in combination with zoledronic acid in a preclinical model of neuroblastoma. Mol. Ther. *21*, 1034–1043.

30. Dieli, F., Poccia, F., Lipp, M., Sireci, G., Caccamo, N., Di Sano, C., and Salerno, A. (2003). Differentiation of effector/memory Vdelta2 T cells and migratory routes in lymph nodes or inflammatory sites. J. Exp. Med. *198*, 391–397.

31. Geginat, J., Lanzavecchia, A., and Sallusto, F. (2003). Proliferation and differentiation potential of human CD8+ memory T-cell subsets in response to antigen or homeostatic cytokines. Blood *101*, 4260–4266.

32. Long, A.H., Haso, W.M., Shern, J.F., Wanhainen, K.M., Murgai, M., Ingaramo, M., Smith, J.P., Walker, A.J., Kohler, M.E., Venkateshwara, V.R., et al. (2015). 4-1BB costimulation ameliorates T cell exhaustion induced by tonic signaling of chimeric antigen receptors. Nat. Med. *21*, 581–590.

33. Beatty, G.L., and Moon, E.K. (2014). Chimeric antigen receptor T cells are vulnerable to immunosuppressive mechanisms present within the tumor microenvironment. OncoImmunology *3*, e970027.

34. Johnson, L.A., Heemskerk, B., Powell, D.J., Jr., Cohen, C.J., Morgan, R.A., Dudley, M.E., Robbins, P.F., and Rosenberg, S.A. (2006). Gene transfer of tumor-reactive TCR confers both high avidity and tumor reactivity to nonreactive peripheral blood mononuclear cells and tumor-infiltrating lymphocytes. J. Immunol. *177*, 6548–6559.

35. Kondo, M., Izumi, T., Fujieda, N., Kondo, A., Morishita, T., Matsushita, H., and Kakimi, K. (2011). Expansion of human peripheral blood γδ T cells using zoledronate. J. Vis. Exp. *55*, 3182.

36. Schilbach, K., Frommer, K., Meier, S., Handgretinger, R., and Eyrich, M. (2008). Immune response of human propagated gammadelta-T-cells to neuroblastoma recommend the Vdelta1+ subset for gammadelta-T-cell-based immunotherapy. J. Immunother. *31*, 896–905.

37. Siegers, G.M., Ribot, E.J., Keating, A., and Foster, P.J. (2013). Extensive expansion of primary human gamma delta T cells generates cytotoxic effector memory cells that can be labeled with Feraheme for cellular MRI. Cancer Immunol. Immunother. *62*, 571–583.

38. De Rosa, S.C., Andrus, J.P., Perfetto, S.P., Mantovani, J.J., Herzenberg, L.A., Herzenberg, L.A., and Roederer, M. (2004). Ontogeny of gamma delta T cells in humans. J. Immunol. *172*, 1637–1645.

39. Kawalekar, O.U., O'Connor, R.S., Fraietta, J.A., Guo, L., McGettigan, S.E., Posey, A.D., Jr., Patel, P.R., Guedan, S., Scholler, J., Keith, B., et al. (2016). Distinct signaling of coreceptors regulates specific metabolism pathways and impacts memory development in CAR T cells. Immunity *44*, 380–390.

40. Zhao, Z., Condomines, M., van der Stegen, S.J.C., Perna, F., Kloss, C.C., Gunset, G., Plotkin, J., and Sadelain, M. (2015). Structural design of engineered costimulation determines tumor rejection kinetics and persistence of CAR T cells. Cancer Cell *28*, 415–428.

41. Nakamura, K., Tanaka, Y., Shitara, K., and Hanai, N. (2001). Construction of humanized anti-ganglioside monoclonal antibodies with potent immune effector functions. Cancer Immunol. Immunother. *50*, 275–284.

42. Thomas, S., Straathof, K., Himoudi, N., Anderson, J., and Pule, M. (2016). An engineered costimulation determines tumor rejection kinetics for more potent and safer cellular therapy of neuroblastoma and other cancers. PLoS ONE *11*, e0152196.

43. Philip, B., Kokalaki, E., Mekkaoui, L., Thomas, S., Straathof, K., Flutter, B., Marin, V., Marafioti, T., Chakraverty, R., Linch, D., et al. (2014). A highly compact epitope-based marker/suicide gene for easier and safer T-cell therapy. Blood *124*, 1277–1287.

YMTHE, Volume *26*

Supplemental Information

Chimeric Antigen Receptor-Engineered Human Gamma Delta T Cells: Enhanced Cytotoxicity with Retention of Cross Presentation

Anna Capsomidis, Gabriel Benthall, Heleen H. Van Acker, Jonathan Fisher, Anne M. Kramer, Zarah Abeln, Yvonne Majani, Talia Gileadi, Rebecca Wallace, Kenth Gustafsson, Barry Flutter, and John Anderson

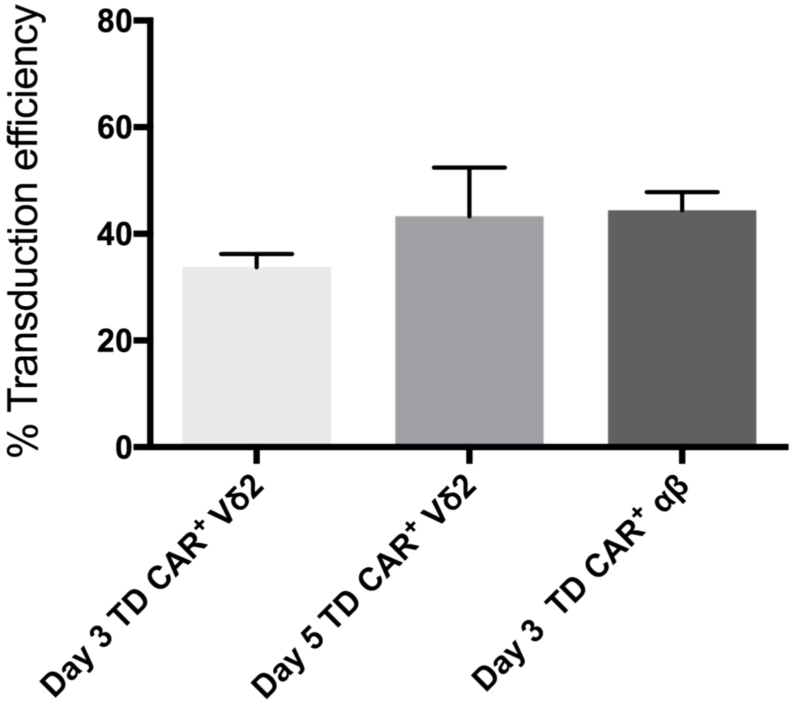

Supplementary Figure 1.
Transduction efficiency of Vδ2$^+$ T cells as measured by % QBend10$^+$ comparing timing of viral transduction (day 3 or day 5). As a positive control PBMC stimulated by CD3 and CD28 antibodies were transduced on day 3 following stimulation.

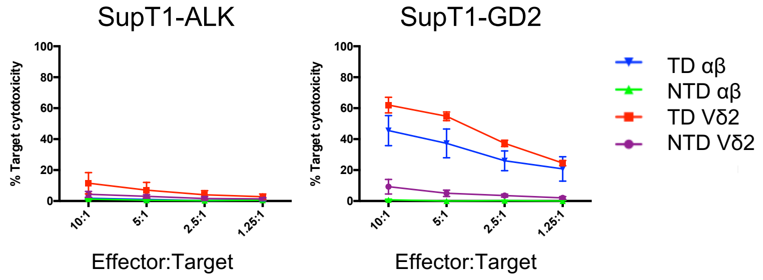

Supplementary Figure 2. αβ and Vδ2⁺ GD2-CAR T cells specifically lyse SupT1 cells genetically modified to express tumor antigen GD2, but not SupT1 cells genetically modified to express anaplastic lymphoma kinase (ALK) antigen in 4-hour ^{51}Cr release assay. GD2-CAR transduced (TD) αβ and Vδ2⁺ T cells were sorted to >95% purity by fluorescence activated cell sorting (FACS). Non-transduced (NTD) cells were sorted by magnetic-activated cell sorting (MACS). Mean of 4 donors in triplicate. Error bars represent SEM.

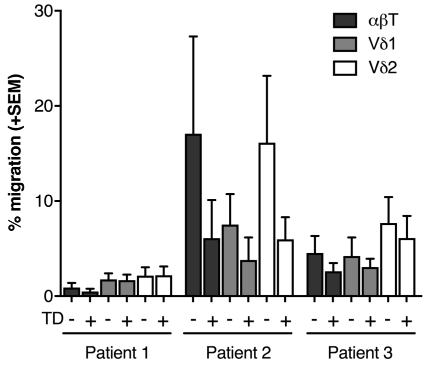

Supplementary Figure 3. Percentage T cell migration towards supernatant taken from three neuroblastoma neurosphere lines derived from primary patient tissue. Migration calculated as ((number of migrated cells in the specific condition - number of migrated cells in the negative control for that condition) / number of migrated cells in the positive control) x 100.
